# Clinical characteristics and outcomes of Hodgkin Lymphoma: A single institution retrospective cohort study

**DOI:** 10.1371/journal.pone.0353363

**Published:** 2026-07-10

**Authors:** Anwar Rjoop, Rania Al-Samama’h, Taima Sari Al-ullemat, Rahaf Alshamali, Batool El-Dwakat, Roaa Al-Saidi, Noor Abdalhakem, Saja Alsarhan, Asmaa Ajarmeh

**Affiliations:** 1 Department of Pathology and Microbiology, Faculty of Medicine, Jordan University of Science and Technology, Irbid, Jordan; 2 Department of Medical Laboratory Science, Faculty of Applied Medical Science, Jordan University of Science and Technology, Irbid, Jordan; 3 Faculty of Medicine, Jordan University of Science and Technology, Irbid, Jordan; Tekirdag Namik Kemal University: Tekirdag Namik Kemal Universitesi, TÜRKIYE

## Abstract

Hodgkin lymphoma (HL) is a highly curable B-cell lymphoma, but a group of patients may experience resistance to initial treatment or progress to refractory disease. Moreover, they may experience relapses or long-term complications such as infertility and secondary neoplasms. In this retrospective cohort study, 257 patients diagnosed with HL between 2002 and 2022 were included to identify clinical and laboratory predictors of survival that may contribute to managing patient treatment and follow-up strategies. Demographic, clinical, treatment, and laboratory data at diagnosis were collected from patients’ e-records. Overall survival (OS) and progression-free survival (PFS) were analyzed using Kaplan–Meier curves and log-rank tests. Univariable and multivariable Cox proportional hazards models were used to identify independent predictors of death. The median age at diagnosis was 29 years, with 54.9% males. Nodular sclerosis was the most common subtype (34.6%). Univariable Cox regression analysis showed that high creatinine levels (HR 7.55, 95% CI 2.62–21.81, p < .001), low platelet count (HR 4.87, 95% CI 1.58–14.94, p = .006), and high total bilirubin (HR 3.76, 95% CI 1.34–10.55, p = .012) are factors associated with poor survival. In the exploratory multivariable Cox regression analysis, poor response to treatment was strongly associated with poor survival (HR 7.89, 95% CI 2.39–26.06, p=<.001), followed by low platelet count (HR 4.72, 95% CI 1.28–17.46, p = 0.020). This may suggest that poor treatment response and low platelet count are independent predictors of poor survival in HL patients but require confirmation in a larger cohort. Early detection of high-risk patients based on these clinical and laboratory results may improve patient treatment and follow-up strategies.

## 1. Introduction

Hodgkin lymphoma (HL) is a less aggressive B-cell lymphoma characterized by Hodgkin/Reed-Sternberg cells in a lymphoid tissue biopsy [1–3]. Subcategorized into two main types, nodular lymphocyte-predominant HL(NLPHL), and classic HL (cHL). cHL is further subclassified based on differences in tumor microenvironment (TME) into four subtypes: Nodular Sclerosis (NS), Mixed Cellularity (MC), Lymphocyte-rich (LR), and Lymphocyte-depleted (LD) [1,2,4]

The worldwide incidence rate for HL in 2020 was 0.98 new cases per 100,000 individuals, resulting in approximately 83,087 cases (age-standardized incidence). High-income countries exhibited the highest incidence rates of HL.

In contrast, the lowest rates were found in low-income countries, such as those in Eastern Asia, South-Eastern Asia, Middle Africa, and Melanesia. In general, males were more affected than females, but sex-based incidence varies heavily depending on the specific type of HL [5]. Females’ incidence is relatively higher for nodular sclerosis, while male predominance is seen in mixed cellularity Hodgkin lymphoma [6].

In Jordan, lymphoma is the fourth most common cancer, and HL is the seventh most common cancer among Jordanian males [7]. According to Aladily et al., HL has a higher incidence rate in Jordan than in other Asian countries [8].

Survival for HL reaches up to 85% of five-year overall survival rates, making it a highly curable disease, especially in younger patients and at early stages of diagnosis (stages I and II), which contributes to favorable outcomes [5,9,10]. Despite this, 5% to 10% of HL patients may experience resistance to initial treatment or may progress to refractory disease [11,12]. Ten to thirty percent of patients experience relapses or long-term complications such as infertility and secondary neoplasms like breast cancer, lung cancer, leukemia, and myelodysplastic syndromes within 5–10 years after treatment, and some patients may develop cardiovascular diseases [5,9,10,13–15].

The International Prognostic Score (IPS) is used to predict the prognosis of advanced-stage Hodgkin lymphoma and consists of seven specific criteria, including age ≥ 45 years, male sex, stage IV disease (Ann Arbor classification), serum albumin < 40g/l, Hb < 10.5g/dl, WBC ≥ 15 × 10³/mm³, and lymphocytes <0.6 × 10³/mm³ or < 8% of white-cell count (lymphocytopenia). In contrast, A-HIPI (Advanced-stage Hodgkin Lymphoma International Prognostic Index) is a new and more accurate prediction model for advanced Hodgkin lymphoma (HL). It was developed by the HoLISTIC Consortium to provide a more individualized risk prediction than the original IPS by using continuous data rather than just “yes/no” cutoffs predicting personalized prognosis [16,17].

This study aimed to identify predictors of advanced stages, overall survival (OS), and progression-free survival (PFS) in Hodgkin lymphoma patients in Jordan. Factors analyzed included demographic characteristics, blood parameters, responses to treatment, and disease stage, in a cohort of HL patients treated at King Abdullah University Hospital (KAUH), a central hospital in northern Jordan.

The results are likely to aid in risk categorization, thereby allowing individualized therapy and improved health outcomes. Ultimately, leading to building a personalized strategy for managing people and whole groups rather than a one-size-fits-all approach.

## 2. Methods

### 2.1. Study cohort, clinical and demographic characteristics

All patients of all ages who were diagnosed with Hodgkin lymphoma between 2002 and 2022 at KAUH were included in the study, for a total of 257 patients. Inclusion criteria: patients with confirmed histopathological diagnosis of HL, diagnosed and treated at KAUH, available pre-treatment clinical and demographic data, and documented follow-up data for survival analysis. Patients were excluded if they were diagnosed outside KAUH. Patients who did not continue their treatment at KAUH, thus having no follow-up data regarding their survival status (alive/died) or treatment outcomes, were excluded from the survival analyses (n = 37). Therefore, only 220 patients who continued their treatment and had follow-up information were included in the survival analyses. Patients’ data, medical history, laboratory blood tests, histopathology results, presenting symptoms, staging, treatment, and outcomes were collected from patients’ e-records (**Table 1**). Diagnosis and subtype classification were done according to the classification of the World Health Organization (WHO). Staging according to the Ann Arbor Staging system (Stage I-IV) [3]. Due to the retrospective nature of the e-records data and patients who did not continue their treatment at KAUH and transitioned to other hospitals, certain baseline prognostic and staging variables., such as ESR, LDH, B symptoms, and PET response (Deauville score), were unavailable and therefore excluded.

**Table 1:** Patients’ Clinical and Demographic Characteristics.

	Number of patients		Percentage(%)
Sample size	Total	257	100
Gender	Male	141	54.9
	Female	116	45.1
Nationality	Jordanian	251	97.7
	Syrian	6	2.3
Diagnosis	NS	89	34.6
	MC	75	29.2
	LR	38	14.8
	LD	4	1.6
	NLP	10	3.9
	Unspecified CHL	41	16.0
Stage	Stage I	21	8.2
	Stage II	88	34.2
	Stage III	62	24.1
	Stage IV	55	21.4
	Not specified	31	12.1
Age groups	(0–15) Years	35	13.6
	(16–35) Years	126	49.0
	(36–45) Years	37	14.4
	+46 Years	59	23.0
Type of treatment	Chemotherapy	195	75.9
	Both	23	8.9
	Not provided	39	15.2
Survival	Died	25	9.7
	Censored (alive, lost, or withdrew)	232	90.3
Response to treatment	Good	172	66.9
	Moderate	14	5.4
	Poor	9	3.5
	Not provided	62	24.1
Relapse	Yes	60	23.3
	No	128	49.8
	Not provided	69	26.8
CNS involvements	Yes	4	1.6
	No	201	78.2
	Not provided	52	20.2
Transformation	Yes	12	4.7
	No	49	19.1
	Not provided	196	76.3
WBC counts	Low	11	4.3
	Normal	141	54.9
	High	92	35.8
	Not provided	13	5.1
Hb levels	Low	95	37.0
	Normal	143	55.6
	High	6	2.3
	Not provided	13	5.1
RBC counts	Low	36	14.0
	Normal	188	73.2
	High	20	7.8
	Not provided	13	5.1
Platelets count	Low	10	3.9
	Normal	163	63.4
	High	70	27.2
	Not provided	14	5.4
Creatinine levels	Low	99	38.5
	Normal	129	50.2
	High	13	5.1
	Not provided	16	6.2
AST levels	Normal	195	75.9
	High	38	14.8
	Not provided	24	9.3
ALT levels	Normal	191	74.3
	High	42	16.3
	Not provided	24	9.3
GGT levels	Low	1	0.4
	Normal	148	57.6
	High	84	32.7
	Not provided	24	9.3
Albumin levels	Low	61	23.7
	Normal	165	64.2
	High	7	2.7
	Not provided	24	9.3
Total bilirubin	Low	71	27.6
	Normal	147	57.2
	High	15	5.8
	Not provided	24	9.3
Family history	Yes	19	7.4
	No	102	39.7
	Not provided	136	52.9
Karyotyping	A mutation cannot be seen	91	35.7
	Abnormal	4	1.6
	Not provided	162	63.0

**NS**: Nodular Sclerosis, **MC**: Mixed Cellularity, **LR**: Lymphocyte-Rich, **LD**: Lymphocyte, **NLP**: Nodular Lymphocyte-Predominant, **cHL**: Classic Hodgkin Lymphoma, **CNS**: Central Nervous System, **WBC**: White Blood Cell, **Hb**: Hemoglobin, **RBC**: Red Blood Cell, **AST**: Aspartate Aminotransferase, **ALT**: Alanine Aminotransferase, **GGT**: Gamma-Glutamyl Transferase

### 2.2. Treatment protocols

Treatment protocols for Hodgkin lymphoma patients included chemotherapy, ABVD (Adriamycin, Bleomycin, Vinblastine, Dacarbazine) as a standard regimen. For early-stage (stage I or II) HL, received in about 2–6 treatment cycles depending on the bulk of disease and the presence of risk factors such as age (more than 45 years), hematological parameters (low hemoglobin, low lymphocytes, or high leukocytes count, serum albumin < 40g/l, and gender (male), stage IV, Hb < 10.5g/dl, WBC ≥ 15 × 10³/mm³, and lymphocytes <0.6 × 10³/mm³. Also, the initial treatment of the early stages is followed by involved-site radiation therapy (ISRT), depending on the case and prognosis [18]. For advanced-stage (stages III or IV) HL patients, ABVD chemotherapy was administered for about 6–8 cycles, and some patients with higher risk received escalated BEACOPP (Bleomycin, Etoposide, Cyclophosphamide, Oncovin, Procarbazine, and Prednisone). Due to the higher pulmonary toxicity for some patients, Brentuximab Vedotin replaced bleomycin in ABVD [19]. For residual or bulky disease, radiation therapy was used. Relapsed/refractory HL patients were administered salvage chemotherapy, followed by autologous stem cell transplant (ASCT) [12].

### 2.3. Outcome characterization

Patients’ response to treatment assessment was done according to the Cheson Criteria, established in 2007 [20]. The overall survival (OS) period for each patient was calculated from the date of diagnosis to the date of death, the last follow-up date, or the last visit to any hospital department available in the patients’ hospital e-records. The progression-free survival (PFS) period for each patient was calculated from the date of diagnosis to the relapse or death date or the last progression follow-up date. For patients with incomplete follow-up dates, where only the year was available, a conservative imputation rule was applied: the date was set to January 1st of that year to calculate OS and PFS. All such cases were considered censored (alive, lost, or withdrew) (n = 16) at this imputed date [21].

### 2.4. Statistical analysis

The study data were statistically analyzed using the Statistical Package for the Social Sciences (SPSS) version 27 (SPSS Inc., Chicago, IL, USA). The data were represented with frequency, percentages, mean, median, and standard deviation (**Table 2**). Continuous variables were summarized as median and interquartile range (IQR). For subgroups with very small sample sizes (n < 5), median and range were reported instead. The data’s normality was assessed using Q–Q plots and Shapiro–Wilk tests; they showed statistically significant deviations from normality (p < 0.05). Statistical analyses were performed to investigate the associations between the clinical features of Hodgkin lymphoma patients (histological subtype and disease stage), their demographic characteristics (age and gender), and laboratory results. Associations between categorical variables and comparisons between groups were assessed using Chi-square, Spearman rho, Kruskal-Wallis, and Mann-Whitney. Blood parameters were categorized based on the reference intervals established by the KAUH Laboratory System (Table A1 in the S1 File). OS and PFS with their potential indicators (age, type of treatment, response to treatment, different blood parameters, and gender, etc.) were analyzed by Kaplan-Meier survival analysis along with a log-rank test (2-sided). Cox regression test (Cox proportional hazards model) to determine significant independent predictors for death and poor survival and find the estimated Hazard ratios (HR) to measure the risk of death by adjusting uni- and multivariable (exploratory). P-value<0.05 (2-sided) was considered statistically significant throughout the analysis.

**Table 2:** Baseline Clinical Characteristics, Laboratory Parameters, and Survival Outcomes of the Hodgkin Lymphoma Cohort.

	Mean	Median	ST.DV
Age at diagnosis (Years)	32.72	29.00	17.47
WBC count (**x10**^**6**^**/mm**^**3**^)	10.97	9.10	8.80
Hb (g/dl)	11.56	11.50	2.52
RBC count (**x10**^**6**^**/mm**^**3**^)	4.57	4.61	1.19
Platelet count (**x10**^**3**^**/mm**^**3**^)	352.31	319.00	152.11
Creatinine level (μmol/L)	62.42	55.00	36.43
AST (U/L)	26.33	19.00	28.62
ALT (U/L)	25.44	15.20	34.15
GGT (U/L)	48.65	25.00	69.77
Albumin (g/l)	40.25	40.10	17.30
Total bilirubin (μmol/L)	12.74	6.50	34.39
Progression Free Survival (Months)	45.02	30.00	43.77
OS (Months)	52.65	35.00	47.36

**ST.DV:** **Standard Deviation, WBC:** **White Blood Cell, Hb: Hemoglobin, RBC: Red Blood Cell, AST: Aspartate Aminotransferase, ALT: Alanine Aminotransferase, GGT: Gamma-Glutamyl Transferase, OS: Overall Survival, g/dl: Grams per deciliter, μmol/L: Micromoles per liter, U/L: Units per liter, g/l: Grams per liter**

### 2.5. Ethical considerations

Ethical approval for this study was obtained before the collection of samples and data from the Institutional Review Board at Jordan University of Science and Technology (JUST) and King Abdullah University Hospital (KAUH) (IRB Approval No. 2023/163/63; dated August 30, 2023). This study was conducted in accordance with the 1964 Helsinki Declaration and its later amendments. Informed consent was waived due to the retrospective and anonymized nature of the samples. Data was accessed on September 10, 2023.

## 3. Results

### 3.1. Population demographics

A total of 257 patients were diagnosed with HL between 2002 and 2022, with a median age at diagnosis of 29 years (range 3–82 years). Males accounted for 54.9% (141/257) of the total group, while females were 45.1%(116/257). The majority of patients were Jordanians, totaling 251 (97.7%), followed by 6 Syrians (2.3%). Eighty-nine (34.6%) patients were diagnosed with NScHL, 75 (29.2%) with MCcHL, 38 (14.8%) LRcHL, 4 (1.6%) LDcHL, 41 (16%) unspecified cHL, and 10 (3.9%) with NLPHL.. The clinical and demographic characteristics of patients are presented in **Table 1**. The patients were categorized into 4 groups based on age to reflect bimodal age distribution for HL 16–35 years and 46 + years age groups; patients in the group (16–35) years are the largest, representing 49% (126), then patients more than 46 years, 23% (59), and patients in ages between (0–15) years and (36–45) years represent 13.6% and 14.4%, respectively.

Normal karyotyping results were observed in 91 patients, while 4 patients showed abnormal findings. Among those with cytogenetic abnormalities, two patients reported a family history of malignancy: one patient with a karyotype of 46, XY [28cells]/46, XY, del(15)(q24)[2cells], had a family history of breast cancer and leukemia, while another patient had 46, XY[16cells]/45, XY[10cells], had a positive family history of colon cancer. No relevant family history of malignancy was noted for the remaining two patients.

A family history of HL was reported in 5/257 patients; the remaining patients had a family history of other malignancies, such as lung cancer, breast cancer, and non-Hodgkin lymphoma (NHL).

### 3.2. Associations Among Clinical, Demographic, and Laboratory Parameters

When all Hodgkin lymphoma subtypes were analyzed together, no statistically significant association between gender and subtype was observed (Pearson chi-square χ²(5) = 10.31, p = 0.067). However, this overall analysis was limited by very low patient numbers in the rarer subtypes, like LDcHL and NLPHL, resulting in 25% of the statistical table cells having an expected count below 5 (as detailed in **Table 3**). Given that NScHL and MCcHL represented the majority of cases (n = 164), a focused subgroup analysis was performed. In this subgroup, where 0% of cells fell below expected counts, females were significantly more likely to have NScHL, (Fisher’s exact, p = 0.030); Odds Ratio [OR] = 2.01, 95% CI: 1.08–3.76) whereas MCcHL predominated among males.

**Table 3:** Distribution of Hodgkin Lymphoma Subtypes by Gender.

		Gender		Total (N)
		Male (n)	Female (n)
Hodgkin Lymphoma Subtype	Nodular Sclerosis	38	51	89
	Mixed Cellularity	45	30	75
	Lymphocyte Rich	27	11	38
	Nodular Lymphocyte Predominant	6	4	10
	Lymphocyte Depleted	2	2	4
	Unspecified CHL	23	18	41
Total		141	116	257

The distribution of HL subtypes did not significantly differ between early-stage (I&II) and advanced-stage (III&IV) patients (p-value = 0.830). Moreover, there was no statistically significant correlation between age and different HL subtypes (Kruskal–Wallis test, H(5) = 10.24, p = 0.069) nor between the type of treatment and disease relapse (Pearson chi-square, p-value = 0.963).

In contrast, a statistically significant association was observed between the response to treatment and subsequent disease relapse (Pearson chi-square χ²(2) = 14.26, p = 0.001). Furthermore, baseline disease stage was significantly associated with relapse status; patients presenting with advanced-stage disease were significantly more likely to experience a relapse compared to those with early-stage disease (Fisher’s exact test, p = 0.016; OR = 2.24, 95% CI: 1.17–4.29).

Hb values were significantly lower in advanced-stage patients (n = 115) than in early-stage patients (n = 106) (Mann–Whitney (U = 4254.5), (Z = −3.88), (p < 0.001); mean ranks of 95.00 vs. 128.36, respectively). Platelet counts demonstrated a significant association with advanced disease, showing higher median levels in Stage III and Stage IV compared to earlier stages (Table 4). Conversely, median creatinine levels (p = 0.556) and total bilirubin levels (p = 0.697) remained statistically uniform across all clinical stages. Furthermore, no significant variations in platelet counts, creatinine, or bilirubin were observed among the different histological subtypes. Thus, these findings suggest that while platelet alterations may reflect advanced disease progression, creatinine and bilirubin operate as independent markers rather than secondary markers of tumor staging.

**Table 4:** Clinical Parameters Stratified by Disease Stage and Histological Subtype.

Stratification Factor	Total (N)	Platelets(x10^3^/mm^3)^ Median [IQR], (n)*	Creatinine(μmol/L) Median [IQR], (n)*	Total bilirubin(μmol/L) Median [IQR], (n)*
**Disease Stage**
Stage I	21	287.5 [70.5], (20)	61.5 [28.0], (20)	6.7 [7.3], (20)
Stage II	88	304.0 [134.0], (86)	59.5 [27.1], (86)	6.6 [4.4], (84)
Stage III	62	362.0 [206.5], (61)	55.0 [30.7], (62)	6.1 [3.7], (62)
Stage IV	55	355.0 [233.0], (53)	53.5 [19.0], (53)	6.3 [5.6], (51)
Unspecified	31	271.0 [183.0], (23)	67.5 [35.5], (20)	7.1 [6.2], (16)
*p-value (Kruskal-Wallis)*	*—*	**0.003**	**0.556**	**0.697**
**Histological Subtype**				
Nodular Sclerosis	89	309.0 [222.0], (87)	54.0 [24.0], (87)	6.0 [4.7], (84)
Mixed Cellularity	75	297.0 [141.5], (70)	61.5 [26.7], (70)	6.5 [5.0], (70)
Lymphocyte Rich	38	359.0 [251.5], (36)	56.5 [32.6], (36)	6.3 [4.3], (34)
Nodular Lymphocyte Predominant	10	337.0 [138.5], (9)	55.0 [25.4], (9)	8.6 [7.0], (9)
Lymphocyte Depleted	4	279.5 [293.3],(4)	57.5 [26.0], (4)	8.7 [5.9-375.0]**, (4)
Unspecified CHL	41	319.0 [134.5], (37)	52.6 [32.0], (35)	6.8 [4.0], (32)
p-value (Kruskal-Wallis)	—	**0.651**	**0.351**	**0.088**

**Note: IQR:** **Interquartile Range, CHL:** **Classical Hodgkin Lymphoma, N:** **Total sample size of the subgroup, n:** **Number of patients with available baseline laboratory records for that specific parameter.**

*Sample sizes vary across laboratory parameters due to missing data or unavailable baseline laboratory records for certain cases.

** Median and range were reported because the sample size was too small for estimation of the IQR. One of the patients in this group presented with secondary biliary obstruction, which explains the profound hyperbilirubinemia (Total Bilirubin = 375.0 μmol/L)

Serum Gamma-Glutamyl Transpeptidase (GGT) levels differed significantly between early and advanced disease stages (Mann–Whitney U = 6961.5, Z = 2.49, p = .013). The mean rank for early-stage patients was 97.41(n = 103) and 118.61 (n = 113) for advanced-stage patients. Also, a significant difference in albumin levels between early-stage (n = 104, mean rank = 125.34) and advanced-stage patients (n = 112, mean rank = 92.87) was observed (U = 4073, Z = −3.815, p < .001).

RBC counts differed significantly between early-stage (mean rank = 120.00, n = 106) and advanced-stage (mean rank = 102.45, n = 115) (U = 5112.0, z = −2.07, p = .038). Mean rank was 120.00 for early-stage patients and 102.45 for advanced-stage patients, indicating that RBC levels were generally lower in advanced-stage disease. White blood cell count (WBC) as categorical variables (low, normal, high), Pearson’s chi-square test showed a borderline difference with disease stage (χ²(2) = 5.97, p = 0.050). (Mann–Whitney, U = 6920, Z = 1.74, p = 0.082).

### 3.3. Survival analyses

#### 3.3.1. Overall Survival (OS), Kaplan-Meier survival analysis.

A total of 220 patients were included in the survival analysis. During the follow-up period, the majority of patients were alive at the last follow-up, with 25 events (deaths), and 195 patients were censored (alive, lost, or withdrew). The median survival time was not reached during the study period. The mean survival time was estimated as 198.9 months (SE = 13.9; 95% CI: 171.7–226.1). Therefore, mean survival estimates should be interpreted cautiously, as they are influenced by censoring. Survival probabilities at specific time points provide a more reliable estimate of outcomes, with a 5-year overall survival of 87.7% and a 10-year overall survival of 83.2%.

**Fig 1:**
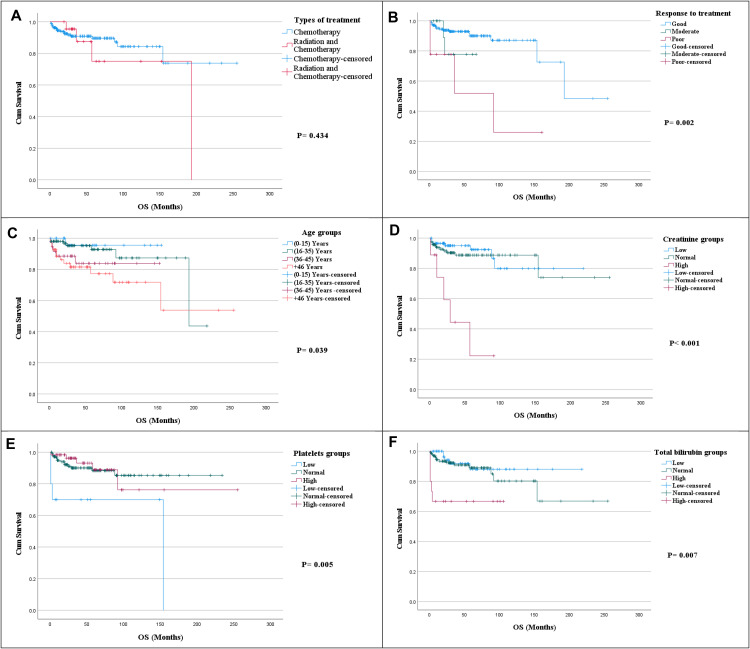
Kaplan–Meier overall survival (OS) curve for the study cohort. The x-axis shows time in months, and the y-axis shows cumulative survival probability. Vertical drops represent death events, and tick marks indicate censored cases**. A.** OS stratified by treatment modality (chemotherapy alone vs. combined radiation and chemotherapy); the log-rank test showed that there is no significant difference in survival between the two groups (p = 0.434). **B.** OS in months stratified by treatment response (good, moderate, and poor). Patients with a good response have significantly longer survival than those with a moderate or poor response (p = 0.002). **C.** OS stratified by the cohort age groups. Younger patients exhibited significantly better survival than those older than 46 years (log-rank p = 0.039). **D.** OS in months stratified by creatinine levels (low, normal, and high). Patients with high creatinine levels demonstrated significantly lower overall survival compared to those with normal or low creatinine levels (log-rank test, p < 0.001). **E.** OS in months stratified by platelet levels (low, normal, and high). Patients with low platelet counts demonstrated significantly lower survival compared to those with normal and high counts (log-rank test, p = 0.005). **F.** OS in months stratified by total bilirubin (TB) levels (low, normal, and high). Patients with high total bilirubin levels demonstrated significantly lower overall survival compared to those with normal or low TB levels (log-rank test, p = 0.007).

Next, the statistical difference in overall survival between patients receiving only chemotherapy and chemotherapy + radiation was studied. The log-rank test showed that there is no significant difference in survival between the two groups (p = 0.434) (Fig 1A). In contrast, the Kaplan-Meier curve of OS and response to treatment showed that patients with a good response had significantly longer survival than those with a moderate or poor response, with a p-value  = 0.002 (Fig 1B). Moreover, better OS was observed in younger patients compared to older patients (> 46 years), (p = 0.039) (Fig 1C).

For blood parameters, patients with high creatinine levels had a low survival time (p < 0.001) (Fig 1D). Additionally, patients with low platelet counts had a lower survival compared to patients with normal and high counts (p-value = 0.005) (Fig 1E). Finally, patients with high total bilirubin (TB) levels had low survival, p-value = 0.007 (Fig 1F).

### 3.4. Progression-Free Survival (PFS), Kaplan-Meier survival analysis

**Fig 2:**
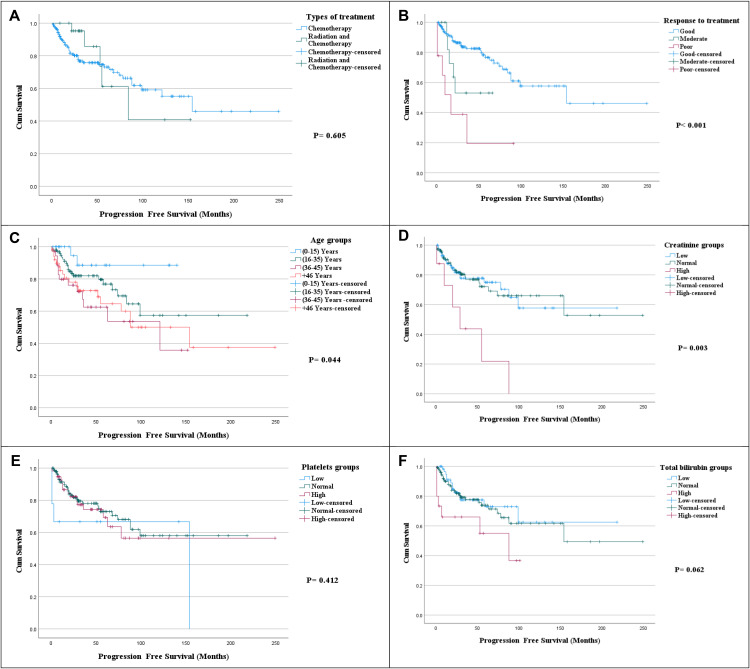
Kaplan–Meier progression-free survival (PFS) curve for the study cohort. The x-axis shows time in months, and the y-axis shows cumulative survival probability. Vertical drops represent events(relapse/death), and tick marks indicate censored cases. **A.** PFS stratified by treatment modality (chemotherapy alone vs. combined radiation and chemotherapy). The log-rank test suggests that the difference is statistically insignificant among the groups (p-value = 0.605). **B.** PFS in months stratified by treatment response (good, moderate, and poor). The curve showed a severe decline in PFS for moderate and poor response patients, log-rank test (p-value<0.001). **C.** PFS is stratified by different age groups. The Log-rank test demonstrates a statistically significant difference in progression-free survival across these age categories (p = 0.044). **D.** PFS in months stratified by creatinine levels (low, normal, and high). High creatinine levels have a statistically significant lower PFS with a log-rank test p-value = 0.003, and they showed a severe decline in PFS in the Kaplan-Meier curve. **E.** PFS in months stratified by platelet levels (low, normal, and high). Platelet levels do not have a statistically significant impact on PFS (log-rank test p = 0.412). **F.** PFS in months stratified by total bilirubin (TB) levels (low, normal, and high). Patients with high total bilirubin levels showed a decline in their survival in the Kaplan-Meier curve, but the log-rank test was insignificant, p = 0.062.

The 5-year progression-free survival (PFS) was 72.4%, while the 10-year PFS was 54.4%. PFS and treatment type analysis: The Kaplan-Meier Curve showed that chemotherapy had better PFS than combined chemotherapy and radiation, but the log-rank test suggests that this difference is statistically insignificant (p = 0.605) (Fig 2A).

After observing the significant relationship between treatment responses and relapse cases above, we wanted to examine its effect on PFS. The PFS for good responders was higher than that of the poor response group. The Kaplan-Meier curve showed a severe decline in PFS for moderate and poor response patients (log-rank test (p < .001)) (Fig 2B).

High creatinine levels had a statistically significant lower PFS, with a log-rank test p-value = .003, and they showed a severe decline in PFS in the Kaplan-Meier curve (Fig 2D). Platelet levels did not have a statistically significant impact on PFS (log-rank test p-value = 0.412) (Fig 2E). Patients with high total bilirubin levels showed a decline in their survival in the Kaplan-Meier curve (Fig 2F), but in the log-rank test, p-value = 0.062.

### 3.5. Cox regression Test (Cox Proportional Hazards Model)

In the univariable Cox regression test, a high creatinine level was a high risk of poor survival (death) (HR 7.55, 95% CI 2.62–21.81, p < .001). Furthermore, low platelet counts had a significantly higher risk of death (HR 4.87, 95% CI 1.58–14.94, p = .006); however, no significant difference in overall survival was observed between high and normal platelet groups (HR 0.85, 95% CI 0.31–2.37, p = .76). Moreover, high bilirubin levels had a significantly increased risk of death (HR 3.76, 95% CI 1.34–10.55, p = .012), but no significant difference in survival was observed between patients with low and normal bilirubin levels (HR 0.69, 95% CI 0.25–1.93, p = .48).

In multivariable Cox proportional hazards analysis, poor response to treatment was the strongest predictor for poor survival from all factors, with a markedly increased hazard (HR 7.89, 95% CI 2.39–26.06, p = 0.001). Low platelet count was also a predictor of increased hazard (HR 4.72, 95% CI 1.28–17.46, p = 0.020). High creatinine showed an association with increased hazard (HR 6.94, 95% CI 1.86–25.94, p = 0.004). While total bilirubin lost its impact.

Given the limited number of events and wide confidence intervals, the number of variables recorded into the multivariable Cox regression was limited to avoid model overfitting. Multivariable analyses were considered exploratory, and primary inferences were based on univariable Cox regression.

## 4. Discussion

This large retrospective cohort study evaluated clinical and laboratory predictors of survival in patients with Hodgkin lymphoma to optimize treatment management and long-term follow-up strategies. Our cohort demonstrated classic epidemiological characteristics, including a young median age at diagnosis, a slight male predominance, and a distinct bimodal age distribution that aligns with established global and local prevalence data [5,7,8,22]. Histological subtyping confirmed nodular sclerosis and mixed cellularity as the primary sub-entities. Interestingly, clear gender-based variations emerged across these subtypes, with female patients exhibiting an increased propensity for nodular sclerosis, while male patients predominated within the mixed cellularity, lymphocyte-rich, and nodular lymphocyte-predominant [6].

A statistically significant association between advanced stage and relapses (p = 0.014) was identified. The advanced stage is characterized by specific hematological and metabolic blood markers that indicate the involvement of high tumor burden and biological immune evasion, which makes standard treatments less effective [19,22–24]. This aggressive disease state is heavily mirrored by the baseline laboratory profiles of our patients.

Specifically, advanced-stage disease is characterized by anemia, indicated by lower hemoglobin and red blood cell counts, alongside marked thrombocytosis, elevated serum GGT, hypoalbuminemia, and leukocytosis. Collectively, these systemic manifestations reflect a state of tumor-induced systemic inflammation, liver stress, poor nutritional reserves, a pro-thrombotic microenvironment, and functional tissue hypoxia [22–24]. These laboratory aberrations highlight biological rationale underlying prognostic scoring systems such as the IPS, which are used to predict disease recurrence.

The median survival time was not reached during the study period because more than half of the cohort remained alive at the final follow-up, preventing an estimation of median OS. The reported mean survival should be interpreted cautiously, as it is sensitive to censoring and follow-up duration. Age at diagnosis emerged as a notable prognostic factor, with younger cohorts demonstrating a better survival profile compared to older patients. Conversely, there was no significant difference between patients receiving only chemotherapy or combination chemotherapy + radiation in OS or PFS. This lack of survival difference based on treatment modality aligns with the findings of Min Lee J. et al. They explored the effect of chemotherapy and radiation therapy and found that neither did influence any significant changes in OS or Event-free survival(EFS) [9]. Furthermore, our observation regarding older age that has poor survival mirrors outcomes described by others, such as those by Rose et al. and Shamoon et al., which highlight the persistent prognostic challenge of managing older HL patients [10,13]. Poor response to initial treatment is a major independent predictor of poor survival, increased mortality, and higher relapse rates in both general cancer and specifically in HL. In Hodgkin lymphoma, failure to achieve a complete remission (CR) after first-line therapy or experiencing early relapse often defines a “primary refractory” status, which carries a significantly higher risk of death [25,26]. Poor initial response necessitates a shift in strategy, often requiring high-dose chemotherapy (HDCT) followed by autologous stem cell transplantation (ASCT) rather than continuing with standard chemotherapy [26]. Furthermore, novel immunotherapy approaches, such as brentuximab vedotin and PD-1 inhibitors, are improving patients’ outcomes [26–29].

Low platelet counts had a lower survival compared to patients with normal and high counts and had a significantly 4.8 times higher risk of death. Several studies have linked thrombocytopenia to worse survival outcomes in breast, lung, gastric, and ovarian cancers; adverse outcomes like bleeding, transfusions, and mortality were more pronounced with lower platelet count levels [30,31]. Moreover, thrombocytopenia is a strong independent predictor of poor OS and PFS in diffuse large B-cell lymphoma (DLBCL) and peripheral T-cell lymphoma (PTCL) [32–34]. Thrombocytopenia may serve as a sign for bone marrow involvement by malignant cells, which subsequently suppresses and replaces normal hematopoiesis [35]. Furthermore, thrombocytopenia could be caused by splenic sequestration; the spleen stores about 30% of the body’s platelets. In an enlarged spleen, this sequestration capacity increases drastically, with 50–90% of platelets being retained in the splenic red pulp [35,36]. Hodgkin lymphoma and other hematological malignancies can cause immune-mediated platelet destruction by autoantibodies or disrupted immune cells, such as T cells and macrophages. In this clinical setting, transient or persistent thrombocytopenia may also be secondary to cancer-associated disseminated intravascular coagulation (DIC) or occult sepsis, which further compounds systemic mortality risk and establishes baseline platelet counts as a versatile marker of poor prognosis. [35,37,38]

Elevated creatinine often reflects structural or functional kidney impairment due to cancer, such as renal infiltration, obstructive uropathy, dehydration, or pre-existing comorbidities like diabetes, hypertension, and heart failure [39–41]. Rapid turnover of tumor cells can lead to tumor lysis syndrome (TLS), causing acute renal failure. In studies, elevated creatinine was associated with significantly worse overall survival in cancers like vulvar and colorectal cancers [42,43]. Furthermore, 10%–25% increases in creatinine over baseline during the first 72 hours of ICU admission or during treatment for cancer patients were associated with doubled hospital mortality [44]. In lymphoma, high creatinine is often caused by renal involvement by the direct tumor cell infiltration or renal side effects of chemotherapy, and it is reported in 26% to 56% of patients [45,46]. While some data suggest that for certain types of lymphoma, low creatinine clearance may not be a significant independent predictor of survival, it still correlates with poor outcomes [42,43,45]. In Hodgkin lymphoma, elevated serum creatinine (SCr) is not a standard risk factor in the IPS; it frequently reflects advanced disease burden or rare complications like renal infiltration [39,47]. The combination of body mass index BMI and SCr has been identified as a survival predictor in patients treated with nivolumab. Renal impairment and elevated SCr often lead to dose reduction of critical agents like platinum-based drugs or methotrexate [39–41,44,47]. Consequently, underdosing to avoid toxicity may result in treatment selection bias, perhaps less effective treatment, and thus lower survival. Therefore, assessing the patient’s renal reserve before starting therapy is critical to understanding the impact of subsequent effects.

This study has several limitations. The retrospective, single-center design may introduce institutional selection bias. Furthermore, the extensive twenty-year study period spans several clinical eras, introducing treatment approach heterogeneity as standard practice guidelines evolved. This retrospective nature also impacted baseline data availability of key prognostic records, such as LDH, ESR, extra-nodal involvement, and PET response (Deauville scores); it prevented a direct, complete comparison of the standard IPS or advanced prognostic models. Finally, the relatively small number of recorded mortality events and high censoring affected survival analyses.

## 5. Conclusion

This study suggests that poor treatment response, creatinine levels, and low platelet count are potential factors associated with poor survival in HL. These findings need validation in a larger cohort. Support should be given to incorporate these potential predictors into routine prognostic assessment to guide individualized management strategies in HL. Early targeted support for high-risk individuals helps prevent serious issues, reduce disease progression, and improve overall population health.

## Supporting information

S1 File Supplementary material. (DOCX)
