# Supplementary material for: Clinical characteristics and outcomes of Hodgkin Lymphoma: A single institution retrospective cohort study
Source: PLoS One. 2026 Jul 10;21(7):e0353363. doi: 10.1371/journal.pone.0353363 (PMC13353956; doi:10.1371/journal.pone.0353363)
Supplement: S1 File — (DOCX) [file pone.0353363.s001.docx]

**Clinical Characteristics and Outcomes of Hodgkin Lymphoma: A Single Institution Retrospective Cohort Study**

Supplementary material

**Table A1.** Blood parameters normal ranges

|  | Low | Normal | High |
| --- | --- | --- | --- |
| WBC (x 10^3^/mm^3^) | <3.5 | 3.5-11 | >11 |
| RBC(x10^6^/mm^3^) | <3.8 | 3.8-5.5 | >5.5 |
| Platelets(x10^3^/mm^3^) | <150 | 150-4000 | >400 |
| HB(g/dl) | <11 | 11-16 | >16 |
| Creatinine(μmol/L) | <53 | 53-97 | >97 |
| GGT(U/L) | <5 | 5-36 | >36 |
| Total bilirubin(μmol/L) | <5 | 5-21 | >21 |
| Albumin(g/l) | <35 | 35-52 | >52 |
| AST(U/L) | - | 0-32 | >32 |
| ALT(U/L) | - | 0-33 | >33 |
